# Methylmalonic acidemia: Neurodevelopment and neuroimaging

**DOI:** 10.3389/fnins.2023.1110942

**Published:** 2023-01-26

**Authors:** Tao Chen, Yian Gao, Shengdong Zhang, Yuanyuan Wang, Chaofan Sui, Linfeng Yang

**Affiliations:** ^1^Department of Clinical Laboratory, Jinan Maternity and Child Care Hospital Affiliated to Shandong First Medical University, Jinan, Shandong, China; ^2^Department of Radiology, Shandong Provincial Hospital Affiliated to Shandong First Medical University, Jinan, Shandong, China; ^3^Department of Radiology, Shandong Yinan People’s Hospital, Linyi, Shandong, China; ^4^Department of Radiology, Binzhou Medical University, Yantai, Shandong, China; ^5^Department of Radiology, Jinan Maternity and Child Care Hospital Affiliated to Shandong First Medical University, Jinan, Shandong, China

**Keywords:** methylmalonic acidemia, diagnosis, nervous system injury, neurodevelopmental characteristics, neuroimaging

## Abstract

Methylmalonic acidemia (MMA) is a genetic disease of abnormal organic acid metabolism, which is one of the important factors affecting the survival rate and quality of life of newborns or infants. Early detection and diagnosis are particularly important. The diagnosis of MMA mainly depends on clinical symptoms, newborn screening, biochemical detection, gene sequencing and neuroimaging diagnosis. The accumulation of methylmalonic acid and other metabolites in the body of patients causes brain tissue damage, which can manifest as various degrees of intellectual disability and severe neurological dysfunction. Neuroimaging examination has important clinical significance in the diagnosis and prognosis of MMA. This review mainly reviews the etiology, pathogenesis, and nervous system development, especially the neuroimaging features of MMA.

## 1. Introduction

Methylmalonic acidemia (MMA), also known as methylmalonic aciduria, is a congenital organic acidemia with multifactorial autosomal recessive inheritance. The disease often causes multisystem injury, especially to the central nervous system (Han et al., 2015). Although the incidence of this disease is low, the mortality rate is very high (Thompson et al., 1989). Estimates of incidence in Western populations range from 1:48,000 to 1:61,000 births for MMA (Manoli et al., 1993). In mainland of China, the morbidity of MMA is about 1:4,000 to 1:26,000 (Zhou et al., 2018). Patients with MMA experience significant mortality, and the prognosis for long term survival is poor. The mortality of mut MMA was 60-88% in the first reports in the 1980s and 1990s and has improved somewhat to ∼40% by the first decade in the 2000s (Zhou et al., 2018). In a study of 88 cblC defects, the mortality rate was 11.4%, and early standard treatment was found to improve biochemical abnormalities, non-neurological signs, and mortality (Fischer et al., 2014). Onset of the manifestations of MMA ranges from the neonatal period to adulthood. If the disease cannot be diagnosed and treated promptly, the financial and psychological burdens on patients, families, and society will be heavy. This study mainly reviews the etiology and pathogenesis of MMA, especially its diagnosis by central neuroimaging, and provides the basis for early diagnosis, early intervention, and improvement of the prognosis.

## 2. Etiology and pathogenesis of MMA

Methylmalonic acidemia (MMA) is the most common type of congenital organic acidemia (Lee and Kim, 2022). It is mainly caused by the deficiency of methylmalonyl-coenzyme A mutase (MCM) or abnormal metabolism of adenosylcobalamin. Under normal circumstances, methylmalonyl-CoA generates succinyl-CoA under the action of MCM and adenosylcobalamin and participates in the tricarboxylic acid cycle. As a result, the conversion of methylmalonyl- coenzyme A to succinyl-CoA is blocked, causing an increase in methylmalonic acid and its associated organic acids, which leads to the abnormal accumulation of intermediate metabolites such as methylmalonic acids, methylmalonyl-CoA, methylmalonyl carnitine, propanoic acid, methylcitric acid, and β- hydroxybutyric acid. The abnormal accumulation of intermediate metabolites causes mitochondrial dysfunction, resulting in multisystem injuries, mainly of the central nervous system (Longo et al., 2005; Obeid and Herrmann, 2006). Brain injuries are the most obvious central nervous system injuries. They mainly manifest as impairments in brain development, brain structure, brain networks, and brain function.

The etiology of MMA is essentially clear. According to the type of enzyme deficiency, MMA can be divided into two categories, MCM deficiency and coenzyme cobalamin metabolism disorder, i.e., mutant type and cobalamin type (Zhou et al., 2018). The mutant type is caused by MCM gene mutations that change the activity of allosteric enzymes. According to the degree of MCM activity reduction, there are two types: mut- and mut0. MCM activity was partially absent in patients with mut- type and completely absent in patients with mut0 type. The degree of MCM activity loss is not only related to the type of MUT gene mutation, but also related to the transcription, translation, protein expression and modification of MUT gene mutation between individuals. The cobalamin type is due to congenital defects in the synthesis, activation or transport of cobalamin (coenzyme of allosteric enzymes), which hinder the synthesis of an effective coenzyme, deoxyadenosylcobalamin, thereby impairing the function of MCM, eventually causing MMA. The cobalamin type can be subdivided into six subtypes (cblA, cblB, cblC, cblD, cblF, and cblH). The mut, cblA, cblB, and cblH types show only MMA and are therefore called isolated MMA; cblC, cblD, and cblF deficiencies are usually associated with homocystinuria, and the resulting condition is called combined methylmalonic aciduria and homocystinuria (MMA-HC). MMA-HC is the main biochemical type of MMA in China, and 60-80% of MMA patients found in China are complicated with homocystinuria (Zhang et al., 2007). Among MMA-HC cases, the cblC type is the most common one (Carrillo-Carrasco et al., 2012).

According to the onset age of MMA patients, MMA can be divided into early-onset MMA (≤ 1 year) and late-onset MMA (>1 year), and early-onset MMA is more common. Different types of MMA patients have symptoms onset at different ages. Most pediatric patients with early-onset MMA develop disease within 1 year and have rapid disease progression, extremely high mortality and morbidity, and poor prognosis; 80% of children with early-onset MMA (those with complete deficiency in allosteric enzyme activity) develop the disease within 1 week of birth, and 90% show clinical symptoms within 1 month of birth; most patients with adenosylcobalamin deficiency or partial deficiency in allosteric enzyme activity show clinical symptoms after 1 month of birth (Matsui et al., 1983). Late-onset MMA patients mostly develop disease between 1 and 3 years of age, with limited symptoms and good prognosis.

## 3. Diagnosis of MMA

### 3.1. Clinical presentation

The clinical manifestations of MMA are diverse and lack specificity, which is related to the type of the disease and the degree of enzyme deficiency. The main clinical manifestations of MMA include feeding difficulty, intellectual disability, psychomotor retardation, ataxia, abnormal muscle tone, seizures, epilepsy, and lethargy (Brismar and Ozand, 1994; Jin et al., 2004). MMA clinical presentations reveal no specific obvious signs and symptoms but rather demonstrate that these conditions affect multiple systems, with the disease often appearing as a fairly multifaceted syndrome, and are age-related (Table 1). In the neonatal period, patients can develop encephalopathy with symptoms such as lethargy, apnea, feeding difficulty, hypotonia, seizures and nystagmus, as well as non-neurological symptoms such as hematological abnormalities, hydrocephalus, and pulmonary hypertension. In infancy, symptoms include vomiting, weight loss, hypoglycemia, visual inattention, neurological deterioration with hypotonia, irritability, and lethargy eventually leading to coma in cases with an acute early-onset neonatal presentation (Forny et al., 2021). Late-onset patients exhibit a wider range of presentations, which can include failure to thrive, various neurological symptoms (encephalopathy, developmental delay, cognitive impairment, epilepsy, ataxia, and pyramidal and peripheral nerve symptoms), and cardiac and kidney manifestations (Grangé et al., 2015; Forny et al., 2021). For patients with MMA, neurological symptoms may be the first symptoms, creating a challenge for physicians in terms of identifying clinical manifestations suggestive of MMA.

**TABLE 1:** Clinical presentation of MMA patients of different ages.

Ages	System	Clinical presentation
Neonates (0–28 days)	Nervous system	Lethargy, apnoea Muscular hypotonia Seizures, neurocognitive impairment Visual inattention or nystagmus Hydrocephalus
	Digestive system	Poor feeding, vomiting
	Blood circulation system	Anemia/thrombocytopenia or pancytopenia Megaloblastosis Cardiomyopathy Pulmonary hypertension
	Urinary system	Hemolytic uremic syndrome
Infants (1–12 months)	Nervous system	Developmental delay (learning disabilities, intellectual disability) Acute encephalopathy Visual and cognitive impairment Microcephaly Muscular hypotonia Seizures Visual inattention or nystagmus
	Digestive system	Anorexia Failure to thrive
	Blood circulation system	Anemia/thrombocytopenia or pancytopenia Megaloblastosis Pulmonary hypertension
	Urinary system	Hemolytic uremic syndrome
Children (1–12 years)	Nervous system	Acute or chronic behavioral or psychiatric abnormalities Acute encephalopathy Movement disorders, dystonia, ataxia, limb weakness Seizures, lethargy Neuropsychiatric disturbance, personality changes Visual impairment (maculopathy, optic atrophy, Peripheral retinopathy) Sensory disturbance
	Blood circulation System	Pulmonary hypertension Venous thrombosis, pulmonary thromboembolism Hypertensive encephalopathy
	Urinary system	Hemolytic uremic syndrome Kidney failure
Adolescence and adulthood (>12years)	Nervous system	Cognitive decline Neuropsychiatric disturbance, personality changes, social withdrawal, depression Decreased ability to study or work Sensory disturbance, ataxia, limb weakness Visual impairment or blindness
	Blood circulation system	Pulmonary hypertension Venous thrombosis, pulmonary thromboembolism Hypertensive encephalopathy
	Urinary system	Hemolytic uremic syndrome Thrombotic microangiopathy Kidney failure

### 3.2. Newborn screening and biochemical detection

Expanded newborn screening for inborn errors of metabolism (IEMs) by tandem mass spectrometry (MS/MS) has enabled simultaneous analyses of more than 40 metabolites and the identification of approximately 50 IEMs during the neonatal period using dry blood spots (Therrell et al., 2015). The levels of propionylcarnitine (C3), C3/acetylcarnitine (C2), and methionine in dry blood spots were measured by MS/MS. When MS/MS screening results exceed the cutoff value and exclude suspected organic acidemia, MMA can be diagnosed by measuring organic acids via gas chromatography (GC)/MS in urine. Increases in methylmalonic acid together with 3-hydroxypropionate and the presence of 2-methylcitrate confirm the diagnosis of MMA (Forny et al., 2021). In China, detection of C3 by MS/MS and detection of organic acids in urine and blood by GC/MS are the preferred methods for the diagnosis of MMA. Newborn screening has been shown to increase the likelihood of early diagnosis of MMA, especially in late-onset cases (Heringer et al., 2016). A follow-up study confirmed significant differences in the incidence rates of intellectual impairment, movement disorders, ocular complication, hydrocephalus, and death when comparing patients with clinical signs before treatment to asymptomatic patients (Ling et al., 2022). When children or adults have atypical manifestations of nervous system damage, MS/MS screening and urinary organic acid examination should be performed promptly to diagnose MMA (Heringer et al., 2016).

Biochemical examination may indicate hyperammonemia, hyperlactemia, metabolic acidosis, hypoglycemia, anemia, urine protein or occult blood positive (Baumgartner et al., 2014). In addition, plasma total homocysteine (tHcy) (rather than free homocysteine) and vitamin B12 were tested to determine whether each case was isolated or combined MMA. We strongly recommend secondary testing using tHcy and MMA to improve specificity and distinguish MMA from other diseases (Huemer et al., 2017).

### 3.3. Molecular genetic analysis

MMA is inherited in an autosomal recessive fashion. To confirm the biochemical diagnosis, guide management, and provide genetic counseling to families, the underlying genetic defect must be identified (Forny et al., 2021). MMA is highly genetically heterogeneous, and at least 10 disease-related genes have been identified; among them, mutations in MUT (mut type), MMAA (cblA type), MMAB (cblB type), MMACHC (cblC type), MMADHC (cblD type), LMBRD1 (cblF type), ABCD4 (cblJ type), and HCFC1 (cblX type) are the most common (Hwang et al., 2021).

Patients of different races or from different regions have varying hotspot mutations. Among combined methylmalonic acidemia cases in China, MMACHC gene mutation is the most common, accounting for approximately 99% of cases, and the most common mutant subtype is c.609G>A (p.W203X), followed by c.80A>G (p.Q27R) and c.658-660delAAG (p.K220del) (Liu et al., 2010; Yu et al., 2021). c.609G>A (p.W203X) and c.567dupT (p.I190fs) are independently associated with poor outcomes, especially for neurodevelopmental deterioration (Ling et al., 2022). In Canada, the most common mutation site in the MMACHC gene is c.271dupA (p.R91KfsX14), where a frameshift mutation accounts for at least 40% of the pathogenic alleles (Morel et al., 2006). However, in isolated MMA, mutation of the MUT gene is the most common, and the most common mutation in China is c.729_730insTT (p.D244LfsX39), accounting for 16.5% of cases (Han et al., 2015). The most common mutations in Brazil and Spain are c.655A>T (p.N219Y) and 322C>T (p.R108C), respectively (Worgan et al., 2006; Randon et al., 2020).

### 3.4. Neuroimaging examination

Neuroimaging in patients with MMA is not routinely performed and often takes place in an emergency situation, especially for those diagnosed by newborn screening who are doing well clinically (Harting et al., 2008). In addition, neuroimaging does not significantly affect clinical decision making in MMA. However, imaging can objectively reflect brain injury conditions, guide diagnosis and treatment, improve the understanding of the disease, and provide an objective basis for early diagnosis and intervention.

Because ultrasound has the characteristics of non-ionizing radiation and low costs, it can also evaluate brain development, degree of brain injury, and neurodevelopmental prognosis. Ultrasound has become a commonly used screening tool for brain examinations of newborns and infants. However, because of the restrictions of imaging technology, the scope of these examinations is limited. Only the brain tissue below the fontanelle can be clearly displayed using ultrasound, and it is significantly less effective than magnetic resonance imaging (MRI) at evaluating the development and extent of the damage to the surrounding brain tissue (Yang et al., 2017). Moreover, ultrasound examination cannot be performed after the fontanelle is completely closed.

The resolution of computed tomography (CT) is relatively high, allowing it to clearly reveal the structure of the brain. However, the brain water content of newborns is relatively high, and the contrast between gray and white matter is not obvious, a situation that is difficult to distinguish from the low density caused by cerebral edema. CT also uses ionizing radiation, which greatly limits the application of CT in the assessment of brain development and brain injury.

Single-photon-emission computed tomography and positron-emission tomography have relatively high sensitivity and can be used to assess brain function and metabolism. However, these two types of examination require the application of radiopharmaceuticals, which can harm newborns and infants, thus limiting their application.

Because of its characteristics of non-ionizing radiation, high tissue contrast, and high spatial resolution, MRI has become the preferred imaging method for newborn or infant brains. The routine brain MRI examination sequences of MMA patients includes T1-weighted imaging (T1WI), T2WI, T2-weighted fluid-attenuated inversion recovery, and diffusion-weighted imaging (DWI). T1WI is mainly used to observe the myelin sheath formation in nerve fibers, giving a high signal. T2WI is mainly used to observe myelin sheath maturation in nerve fibers, giving a low signal. T1WI is mainly done before 6 months of age, and T2WI is mainly done after 6 month of age. Therefore, MRI can be used to observe the formation of a normal myelination process, delayed myelination, or myelination hypoplasia and to evaluate brain development. In particular, MRI functional brain imaging and other technologies can comprehensively evaluate the brain development and brain injury degree of MMA patients, and make qualitative diagnosis, provide objective basis for clinical diagnosis, help to judge prognosis and improve the quality of life of patients.

## 4. Mechanism of nervous system injury and neurodevelopmental characteristics

### 4.1. Mechanism of nervous system injury

Nervous system injury is the main injury caused by MMA (Cudré-Cung et al., 2016). The pathophysiological mechanism of nervous system injury involves inhibition of succinate dehydrogenase by high concentrations of methylmalonic acid, which is necessary for aerobic oxidation of mitochondrial glucose. High concentration of organic acid metabolites such as methylmalonic acid, propionic acid and 2-methylcitric acid lead to mitochondrial dysfunction and further lead to neuronal cell apoptosis (Jafari et al., 2013). The globus pallidus is the most sensitive to mitochondrial dysfunction and is often the first site of injury. In MMA-HC, methionine synthase is obstructed, resulting in blocked methylation, homocysteine accumulation, and S-adenosinemethionine reduction, the deficiency of which is associated with dysmyelination (Rossi et al., 2001). The role of myelin sheath is to protect neurons and transmit the nerve impulse to the neuron, therefore, the dysmyelination can affect the conduction of nerve impulse, triggering a series of nerve damage. Furthermore, children with congenital succinate dehydrogenase dysfunction often present with globular lesions, and an increased lactate content compensates for mitochondrial energy metabolism disorders (Okun et al., 2002). In addition, brain development, including ganglioside-mediated regulation of synaptic plasticity, is also impaired, and cognitive and behavioral changes will occur (Kölker et al., 2003). Current studies mainly focus on the enhancement of oxidative stress and mitochondrial dysfunction caused by MMA, and most of these theories are involved in the nervous system injury of MMA through neuronal apoptosis. This has also been confirmed in animal studies, high concentrations of methylmalonic acid could significantly alter the morphology of rat cortical neurons, attenuate cell viability and aggravate cell apoptosis. Enrichment analysis of the apoptosis-related genes revealed that the MAPK and p53 signaling pathways may be involved in the pathogenesis of MMA (Han et al., 2015). Unfortunately, even if the majority of MMA patients are treated promptly, there will be some degree of neurological damage, residual mental retardation, motor and language delays and other complications (Shea et al., 2012).

### 4.2. Neurodevelopmental characteristics

#### 4.2.1. Cognitive impairments

Many MMA patients suffer from cognitive impairments (Molema et al., 2021). Cognitive impairment occurs through neuroinflammatory and stress-induced damage pathways during the process of neuroinflammation and oxidative stress injury caused by MMA (Gabbi et al., 2017; Li et al., 2021). O’Shea et al. adopted the age-appropriate Wechsler Intelligence Scale to evaluate the neuropsychological results of 43 patients with simple MMA (including the mut type, cblA type, and cblB type) and reported significant differences in neurocognitive outcomes among patients (Shea et al., 2012). Intelligence is affected by the MMA subtype and onset age, and patients with early-onset MMA exhibit the most severe neurocognitive impairment, with even greater impairment in patients with a history of hyperammonemia and seizures at diagnosis. Children with this disorder are at risk for developmental delays, cognitive difficulties, and progressive declines in function. With the exception of patients with the enzyme-deficient cblA subtype, the mean IQ of the patient population including all types is rarely above 85, while the mean IQ of the patient population with the mut0 enzyme subtype is considerably lower (Waisbren, 2022). A clinical cohort study reported that 12 children with cblC defects diagnosed by newborn screening were found to have developmental delays, most of whom presented with hypotonia and nystagmus, while neurodevelopmental assessments suggested that impaired motor development was the most prominent deficiency (Weisfeld-Adams et al., 2013). Late-onset patients may have atypical symptoms such as mental symptoms and cognitive decline (Hwang et al., 2021).

#### 4.2.2. Intellectual and motor developmental disorders

The clinical symptoms of early onset MMA are mainly neuropsychiatric injury, common developmental lag, abnormal mental behavior, hydrocephalus, epilepsy and so on. Neurological impairment most commonly manifests as movement disorders, such as involuntary tremor, gait instability, dystonia, hypotonia, muscular rigidity and chorea (Mink, 2003). For the developmental follow-up of MMA patients, the developmental quotient (DQ) can be evaluated from five aspects: adaptive behavior, gross motor, fine motor, language, and personal social behavior. Intelligence quotient (IQ) can also be used to evaluate intelligence. It has also been demonstrated that serum methylmalonic acid, cytokines, reactive oxygen species (ROS) and reactive nitrogen species (RNS) cross the blood-brain barrier, cause cognitive impairment, and are positively correlated with DQ and IQ scores (Li et al., 2021). MMA can lead to nervous system injury, resulting in varying degrees of intellectual and motor retardation, seriously affecting the quality of life of children. A follow-up study in China used a pediatric neuropsychological development examination scale for 0- to 6-year-old patients [China neuropsychological and Behavior Scale-Revision 2016 (CNBS-R2016) scale, Capital Medical University] (Fischer et al., 2014). This scale mainly tests the physical abilities and intelligence of infants and children in five aspects: gross movements, fine movements, adaptability, language, and social behavior. After standardized treatment, the DQ and subscale scores increased in most of the patients, although these differences did not reach significance.

#### 4.2.3. Visual damage

Long-term neurological damage may be present in the visual system. In particular, optic atrophy is an increasingly recognized late presentation of MMA, resulting in acute or chronic vision loss (Alvarez et al., 2016). In patients with MMA, it can also show macular disease, peripheral retinopathy, nystagmus, strabismus, optic atrophy, etc. (Gizicki et al., 2014).

#### 4.2.4. Seizures

Seizures are also a recognized symptom of MMA and may occur at the time of onset or later (Kölker et al., 2015). Seizures may present tonic-clonic seizures, tonic seizures, clonic seizures and partial motor seizures. The electroencephalo-graph and seizure patterns are non-specific. Severely affected patients may be difficult to treat and may have recurrent status epilepticus.

## 5. Imaging findings of the central nervous system in MMA patients

Some new functional MRI technologies have gradually emerged, including susceptibility-weighted imaging, high-resolution 3D-T1WI, magnetic resonance spectroscopy (MRS), Positron Emission Tomography/Magnetic Resonance (PET/MR) and diffusion tensor imaging (DTI), diffusion kurtosis imaging, neurite orientation dispersion and density imaging, and resting-state functional connectivity MRI. Some studies have shown that the lesion area is characterized by decreased acetylaspartate (NAA) level and increased lactate (Lac) level in MRS, while the creatine (Cr) and choline (Cho) levels in other brain areas are normal, after treatment, the levels of Lac and Cr decreased, the level of NAA decreased, and the clinical symptoms gradually improved, so it can be used for non-invasive detection of treatment effect (Takeuchi et al., 2003). Lac was not detected in normal brain parenchyma and cerebrospinal fluid, and elevated Lac levels were detected in some cerebrospinal fluids. PET/MR brain scanning showed that the uptake of 18F-FDG in bilateral caudate nucleus and putamen increased, the uptake of 18F-FDG in frontal lobe decreased, and the extracerebral space widened in the early stage. The decreased uptake of 18F-FDG in frontal lobe reflected the atrophy and volume reduction of frontal lobe. The uptake of 18F-FDG in the caudate nucleus and putamen decreased in the late stage (al-Essa et al., 1999). Studies have shown that DTI has the potential to quantify nerve fibers and is more sensitive than conventional MRI in evaluating brain development and brain injury. However, these new technologies have not been widely used in routine MRI examinations for patients, only part of functional imaging has been gradually applied to the research of MMA. MRS has a relatively large number of cases in the study of MMA, but the number of cases in different literatures (Takeuchi et al., 2003; Michel et al., 2004) is small, and large-sample literature support is needed. Some studies have found that functional changes confirmed by PET/MR are greater than morphological abnormalities shown by MRI, and have better correlation with the clinical course of the disease; There is great potential in the assessment of neurodegenerative diseases in children; However, caution should be exercised when extrapolating data from individual case reports and further detailed studies are therefore required. There is also evidence that DTI is a more sensitive technique for evaluating nerve fibers (Gao et al., 2009). DTI has great potential for brain evaluation in children with MMA, but one of the challenges is to determine the size and shape of the scan field. However, at present, the number of cases involved in various functional imaging is relatively small, and its representativeness needs further research. With the continuous improvement of the above techniques, we believe that these new technologies can be gradually applied in the imaging diagnosis of MMA in the near future.

Patients with early-onset MMA are characterized by brain retardation or dysplasia and brain tissue damage. The main manifestations include decreased white matter volume, widened extracerebral space, cortical atrophy, delayed myelination or myelination hypoplasia, thin corpus callosum, widened ventricular system, and symmetrical abnormal signals of the basal ganglia (especially the globus pallidus) (Michel et al., 2004; Radmanesh et al., 2008). Myelination retardation or dysplasia showed white matter isointensity and hypointensity on T1WI and white matter hyperintensity on T2WI and T2 FLAIR, which distributed around the lateral ventricle, partially confined to the lateral ventricle triangle or the posterior part of the semioval center, and distributed symmetrically on both sides. The corpus callosum was thin and hyperintense on T2WI. In acute phase, globus pallidus showed symmetrical hypointensity on T1WI, hyperintensity on T2WI and T2FLAIR, hyperintensity on DWI and hypointensity on ADC. The result showed that that globus pallidus increase in early stage, decreased in late stage, and finally showed hypointensity on T1WI, hyperintensity on T2WI and hypointensity on T2FLAIR (Trinh et al., 2001; Burlina et al., 2003). The imaging findings in patients with late-onset MMA also include anomalies such as spinal cord atrophy (Martinelli et al., 2011). The pathological changes associated with late-onset MMA are mainly characterized by multifocal demyelination in the brain and spinal cord. The lesions were isointense or hypointense on T1WI and hyperintense on T2WI and T2 FLAIR (Bodamer et al., 2001; Smith et al., 2006; Tsai et al., 2007; Thauvin-Robinet et al., 2008) (Table 2).

**TABLE 2:** Imaging manifestations of MMA.

Early-onset MMA	From a whole point of view	The volume of white matter decreased, the sulci of cerebrum and cerebellum deepened, and the cortex atrophied		
	From a local point of view	Myelination retardation or dysplasia	The white matter was isointense or hypointense on T 1WI and hyperintense on T 2WI and T 2 FLAIR. The white matter was distributed around the lateral ventricle, partly confined to the lateral ventricle triangle or the posterior part of the semioval center, and distributed symmetrically on both sides	
		Corpus callosum	In addition to thinning, hyperintensity was also observed on T 2WI	
		Globus pallidus	Acute phase	Symmetrical hypointensity on T1WI, hyperintensity on T2WI and T2FLAIR, hyperintensity on DWI and ADC were observed
			Dynamic follow-up	The volume of globus pallidus increased in the early stage and decreased in the late stage, and finally showed cerebrospinal fluid signal intensity, which was hypointensity on T1WI, hyperintensity on T2WI and hypointensity on T2FLAIR
Late-onset MMA	In addition to that above manifestation, late onset MMA also includes spinal cord atrophy and other abnormal change. Its pathological changes are mainly characterized by multifocal demyelination in the brain and spinal cord, which is isointense or hypointense on T1WI and hyperintense on T2WI and T2 FLAIR			

The most common imaging manifestation in the brain of MMA patients is brain atrophy (Chen, 2008; Radmanesh et al., 2008), manifested as varying degrees of deepening cerebral and cerebellar sulci, cortical atrophy, and enlargement of the extracerebral space. The cause of brain atrophy is the decrease in the cerebral white matter volume, that is, the developmental delay of the white matter or white matter hypoplasia. Brain atrophy can directly affect the development of the brain and nervous system in newborns or infants.

Diffuse supratentorial white matter swelling in MMA-HC patients is common, and white matter swelling can cause white matter damage that involves U-shaped fibers (Weisfeld-Adams et al., 2013). The abnormal MRI signals in white matter may be related to edema, developmental delay, and hypoplasia of myelin. Some scholars believe that the pathophysiological changes of white matter are mainly vascular injury, which may be related to poor methylation and accumulation of non-physiological fatty acids (Rossi et al., 2001).

The brain myelination hypoplasia of MMA patients is usually located in the anterior and posterior horns of bilateral lateral ventricles and the periventricular white matter and semioval center, showing a symmetric distribution, which are different from demyelinating lesions. Brain myelination hypoplasia is associated with deficiency of S-adenosylmethionine in the brain caused by metabolic abnormalities (Weisfeld-Adams et al., 2013). If the dysplastic myelin sheath cannot be diagnosed and effectively treated at an early stage, it will result in low white matter volume, leading to a poor prognosis. A thin corpus callosum is common in children with low white matter volume and is a response to the decrease in white matter volume. It may be congenital or acquired. The abnormal MR signals of the bilateral lateral paraventricular nucleus and semioval center white matter and the thin corpus callosum may all be associated with delayed myelination or myelination hypoplasia (Yes̨ilda et al., 2005).

Hydrocephalus is also common in MMA patients. Some scholars have suggested that its formation mechanism may be related to increased cerebrovascular stiffness, which results in decreased arterial compliance, increased arterial pressure, no decay of arterial pressure, and increased intracranial pressure, eventually causing hydrocephalus (Greitz et al., 2010). High concentrations of cysteine metabolites have toxic effects on the vascular wall, which is the main cause of vascular endothelial damage (Herrmann and Knapp, 2002).

The basal ganglia (especially the globus pallidus) are the most frequently involved sites in MMA patients. The mechanism of basal ganglia involvement may be related to the reduced activity of cytochrome C oxidase and succinate dehydrogenase, resulting in the accumulation of toxic organic acid metabolites, leading to damage to the basal ganglia, especially the globus pallidus, which has a relatively high energy demand (Michel et al., 2004; Baker et al., 2015; Fraser and Venditti, 2016). During the acute exacerbation of MMA patients, the globus pallidus shows low density on plain CT images; MRI manifests as a low T1WI signal, high T2WI signal, high DWI signal, and apparent diffusion coefficient graphs with a low signal with symmetrical distribution; MRS shows an increase in the lactate peak and a decrease in the N-acetylaspartic acid (NAA) peak. During metabolic decompensation, there is an abnormally high signal on DWI, suggesting infarction, which Trinh et al., 2001 believed is related to cell membrane rupture caused by mitochondrial dysfunction with accumulation of lactic acid. After a reasonable drug treatment, the abnormally high signal on DWI can gradually disappear and return to normal, the blocked diffusion subsides, and the lactate peak decreases. Therefore, many scholars believe that an abnormally high DWI signal reflects some cytotoxic edema. The persistently abnormally high signal intensity of DWI suggests nervous system injury and poor prognosis. Although the abnormal signal in the basal ganglia (especially globus pallidus) is a relatively specific manifestation in MMA patients, the incidence of this sign is not very high (Krishna et al., 2008).

DWI has been used to investigate the apparent diffusion coefficient (ADC) in MMA, which has been reported to be associated with restricted water diffusion in the globus pallidus that may resolve after clinical intervention, including carnitine supplementation. Findings from 12 MMA patients demonstrated significant reductions in fractional anisotropy (FA) in the globus pallidus and frontal, temporal, and occipital white matter on diffusion tensor imaging (DTI), which were not observed on conventional T1/T2 images (Gao et al., 2009), suggesting that in addition to restricted water diffusion in the globus pallidus, MMA is associated with more widespread disturbances in white matter integrity. The referenced study serves as an example of how DTI has been demonstrated to be superior to routine imaging in identifying these diffuse lesions. Neurocognitive lesions in MMA have yet to be investigated with fMRI (Gropman and Anderson, 2020).

In summary, with the rapid development and innovation of gene technology, MMA patients have received increasing attention in clinical practice. Early detection and early diagnosis using imaging examinations are particularly important. In particular, brain functional imaging by MRI and other technologies can comprehensively assess the brain development and the degree of brain damage in MMA patients and lead to a qualitative diagnosis. Because MRI evaluation and qualitative diagnosis can provide an objective basis for the clinical diagnosis, it can help determine the prognosis and improve the quality of life of patients.

## 6. Conclusion

This paper reviews the etiology, pathogenesis, diagnosis, neurodevelopmental features, and recent advances in neuroimaging studies of MMA. MMA is a serious disease with a complex clinical phenotype and genotype and is difficult to identify from signs and symptoms, thus necessitating biochemical tests. Newborn screening is of marked significance and is the key to early detection and treatment. Accurate diagnosis is the basis of accurate treatment. Given the various forms of the disease, misdiagnosis and missed diagnosis are not uncommon. For children with low intelligence, abnormal behavior, general convulsions, hypertonia or hypotonia, symmetrical limb paralysis, or polyneuropathy as the main symptom in combination with imaging characteristics, the possibility of MMA should be considered regardless of whether a positive family history has been identified. Early diagnosis and treatment of MMA are essential for improving the prognosis and quality of life of patients.

## Author contributions

TC and YG drafted the manuscript for publication. SZ prepared the tables. YW participated in writing chapter etiology and pathogenesis and diagnosis of MMA. CS participated in writing chapter neuropsychology and neuroimaging of MMA. LY reviewed and revised the manuscript. All authors contributed to the article and approved the submitted version.
